# Triacylglycerol metabolism is a novel target to combat West Nile virus infection

**DOI:** 10.1080/22221751.2026.2728216

**Published:** 2026-09-01

**Authors:** Flavia Caridi, Patricia Mingo-Casas, Ana Esteban, Teresa Poderoso, Nereida Jiménez de Oya, Eva Calvo-Pinilla, Bo Zhang, Eva-María Priego, María-Jesús Pérez-Pérez, Ana-Belén Blázquez, Miguel A. Martín-Acebes

**Affiliations:** a Department of Biotechnology, Instituto Nacional de Investigación y Tecnología Agraria y Alimentaria, Consejo Superior de Investigaciones Científicas (INIA-CSIC), Madrid, Spain; b Centro de Investigación en Sanidad Animal (CISA INIA-CSIC), Valdeolmos, Spain; c State Key Laboratory of Virology and Biosafety, Chinese Academy of Sciences, Wuhan Institute of Virology, Wuhan, People’s Republic of China; d Instituto de Quimica Medica (IQM, CSIC), Madrid, Spain

**Keywords:** West Nile virus, lipid, triacylglycerol, antiviral, neuroinflammation

## Abstract

West Nile virus (WNV) is a zoonotic *Orthoflavivirus* transmitted by mosquitoes that is responsible for outbreaks of meningitis and encephalitis worldwide. Driven by climate change, WNV has expanded as a global public health concern, particularly in temperate regions. However, there are still no specific approved therapies, reinforcing the need for antiviral development. Previous works have documented that WNV multiplication strictly depends on certain cellular lipids. To identify novel lipid-related therapeutic targets, we analyzed the infection-driven alterations in the CNS lipidome, the primary tissue supporting WNV replication. Our results indicated that the major alterations in the brain lipid content of WNV-infected mice corresponded to triacylglycerols (TAGs). Moreover, transcriptomic analysis showed that infected brains underwent changes in the expression of TAG metabolism. Supplementation with exogenous fatty acids increased lipid droplets (LD) content and promoted viral replication in cell culture models. On the contrary, pharmacological intervention in TAG metabolism using diacylglycerol acyltransferase inhibitors (DGATi) suppressed WNV multiplication in cell culture models. As a proof-of-concept of the therapeutic potential of DGATi, treatment of mice with A922500 reduced viral burden in the brain and proinflammatory cytokine production. Overall, our results unveil the importance of LDs and glycerolipid metabolism for WNV and highlight the potential of therapeutic interventions targeting this pathway to control viral replication and neuroinflammation.

## GRAPHICAL ABSTRACT

**Figure:**
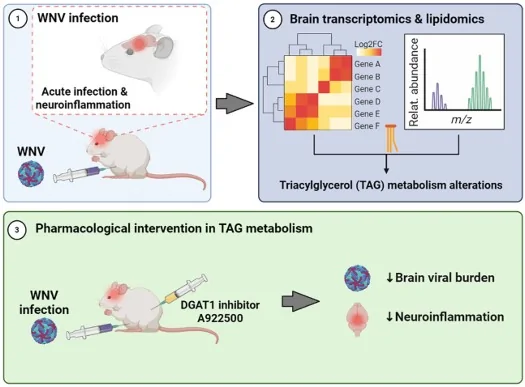

## Introduction

West Nile virus (WNV) is a zoonotic mosquito-borne pathogen classified into the genus *Orthoflavivirus* from the family *Flaviviridae* [1]. This virus circulates among birds, which act as its natural reservoir, and can spill over into humans through the bites of infected mosquitoes [2]. Over the last decades, WNV has emerged as a global public health concern, particularly in temperate regions of North America, Europe, and the Mediterranean basin [3]. Its expansion is driven by a complex interplay of factors including avian hosts, globalization of travel and trade, land use, competent vector abundance, population density, viral adaptation, and, most notably, climate warming [4-6]. Milder winters and longer warm seasons with increased mosquito activity facilitate overwintering of the virus within mosquito populations, year-to-year transmission, and establishment of endemic cycles [3]. Approximately 80% of human WNV infections are asymptomatic, but 20% of them cause a febrile illness named West Nile fever (WNF), and less than 1% cause WNV neuroinvasive disease (WNND). Severe cases may present with meningitis, encephalitis or acute flaccid paralysis, and can be fatal or result in long-lasting neurological sequelae [7,8]. Despite its clinical relevance, there are no approved or recommended therapies for WNV and management is limited to supportive care. Given the current eco-epidemiological scenario and the increasing incidence of WNV infection, the identification and evaluation of new therapeutic strategies have become essential.

WNV infection strictly depends on cellular factors to complete the virus life-cycle, including certain lipids [9]. Viral factories and *de novo* particle biogenesis take place at virus-induced organelle-like membranous structures with specific lipid composition. Cholesterol, fatty acids, sphingolipids, and certain glycerophospholipids play important roles in WNV multiplication [10-14]. Consequently, modulation of lipid metabolism is being explored as an alternative strategy for identifying antiviral therapeutics. This approach is further supported by the antiviral efficacy of approved and investigational agents that target lipid metabolic pathways and have demonstrated clinical utility in cardiovascular disease, metabolic disorders, and certain types of cancer [15,16]. Data from *in vivo* infection models have confirmed that altering the cellular contents of specific lipids, such as fatty acids or sphingolipids, can modulate viral replication and neuroinflammation caused by WNV [17,18]. This host-targeted strategy may have potential advantages over classical direct-acting antivirals targeting viral enzymes (*e.g.* polymerases or proteases). These advantages arise from their potential broad-spectrum activity against related viruses that exploit shared host cellular factors, their prospective efficacy against future emerging pathogens, and their inherently higher genetic barrier to the development of antiviral resistance [19].

In this work, we have analyzed the impact of WNV infection on the brain lipidome in a mouse model, leading to the identification of triacylglycerols (TAGs) as the major altered lipid species. Pharmacological interventions on TAG metabolism reduced viral burden in the brain and associated neuroinflammation, providing a proof-of-concept for the *in vivo* druggability of this metabolic pathway to treat WNND.

## Materials and methods

### Ethics statement

Animals were handled in strict accordance with the guidelines of the European Community 86/609/CEE and the Spanish Animal Welfare Law 32/2007. Procedures were approved by the CSIC Ethics Committee and authorized by the Division of Animal Protection of the Comunidad de Madrid (permits numbers PROEX 104.2/21 and PROEX 155.4/24).

### Animal experiments

A total of 16 six-week-old C57BL/6JOlaHsd (Inotiv) female mice (8 WNV-infected and 8 mock-infected) and 22 six-week-old female Hsd:ICR (CD-1) mice (Inotiv) (all infected) were used. Female mice were selected to limit experimental variability and to facilitate stable group housing by minimizing aggressive behavior and associated stress, thereby improving experimental standardization and animal welfare. For lipidomics experiments, we selected C57BL/6J inbred mice to enable easier interpretation of experiments using a reduced number of mice and in order to allow identification of potential therapeutic targets with higher confidence than using outbred strains. On the contrary, CD-1 outbred mice, which have more genetic diversity, better resembling natural population variability, were selected for validation of therapeutic targets. Mice were infected with 1 × 10^4^ plaque forming units (PFU) of WNV NY99 (GenBank: KC407666.1) diluted in 200 µl of Minimum Essential Medium Eagle (MEM; Corning) by intraperitoneal (i.p.) injection, and mock-infected mice were inoculated i.p. with the same volume of MEM [20,21]. For drug treatment experiments, infected-mice were randomly divided into two groups of eleven animals treated with drug vehicle (vehicle group) and eleven animals treated with DGATi A922500. DGATi or its vehicle alone (1% Tween-80 in PBS) was orally administered at a dose of 3 mg/kg with animal-feeding curved needles (20 g × 1.5″, 2.25 mm, Cadence Science). Mice were daily treated with the drug or vehicle starting the day before infection up to day 6 post-infection. Animals were kept with *ad libitum* access to food and water during the whole experiment and were anesthetized under isoflurane and euthanized at 7 days post-infection for sample collection. All samples were immediately frozen and stored at −80 °C until further analyses.

### Viral infections in cell culture

Infectious virus manipulations were conducted in biosafety level 3 facilities. Vero CCL-81 (ATCC) and Neuro 2A (ATCC CCL-131) cells were cultured in MEM (Corning) and DMEM (Gibco) respectively supplemented with penicillin/streptomycin, 2 mM L-glutamine, and 10% fetal bovine serum. Cells were pre-treated for 1 h with A922500 and AZD3988 (MedChem Express) or the same amount of drug vehicle and infected with WNV NY99 at a multiplicity of infection (MOI) of 1 plaque forming units (PFU)/mL. After 1 h of incubation at 37°C, the viral inoculum was removed, and fresh medium supplemented with 2% fetal bovine serum and containing A922500, AZD3988, or DMSO was added. Infections were carried out for 24 h at 37°C. The viral progeny released into the culture supernatant was titrated by standard plaque assay in semisolid agarose medium [22]. Uninfected cells were treated in parallel, and the amount of cellular ATP was determined using CellTiter-Glo Luminescent Cell Viability Assay following the instructions provided by the manufacturer.

### Recombinant viral particle (RVP) assays

Single-cycle infectious RVP production was performed by double transfection of HEK-293 T cells (CRL-1573, ATCC) of a plasmid pcDNA 3.1(+) encoding a codon optimized version for mammalian expression of WNV NY99 structural proteins (C, prM and E) together with a CMV-driven subgenomic replicon encoding WNV NY99 genomic sequence with the structural genes replaced by Renilla luciferase [23]. WNV RVPs bearing a fluorescent reporter were produced as previously described [20]. RVPs were harvested at 48-72 h post-transfection. Vero cells were pretreated with DGAT inhibitors A922500, AZD3988 for 1 h, or sodium oleate (MedChemExpress) complexed with 5% fatty acid-free bovine serum albumin (BSA), and infected with RVP in the presence or in the absence of the inhibitors or the respective vehicle controls (DMSO for DGATi and BSA for sodium oleate treatment). In the case of sodium oleate, the compound was only present during the 24 h pretreatment period. Viral replication was measured by determination of luciferase activity using Luciferase Assay System TM055 (Promega) at 48 h post-infection.

### Virus-like particle (VLP) assay

VLP production was quantified using HeLa cells stably expressing WNV structural proteins [24] as previously described [17]. Cells were treated with DMSO or 50 µM A922500 for 20 h; culture medium was replaced with fresh medium containing the corresponding treatment, and incubation was continued for an additional 4 h. Supernatants collected during this period, containing newly released VLPs, were harvested and analyzed by enzyme-linked immunodot assay using mouse monoclonal antibody MAB8150 against the WNV envelope protein (EMD Millipore) and appropriate secondary antibody coupled to horseradish peroxidase. Signals were detected by chemiluminescence and acquired using a ChemiDoc XRS Imaging System (Bio-Rad).

### LD staining

Cells were washed once with serum-free MEM and stained with 10 µM Nile Red (ThermoFisher) for 30 min at room temperature. Cells were then washed three times with serum-free MEM, fixed with 4% paraformaldehyde, mounted, and analyzed by fluorescence microscopy. Cells were imaged at 60x magnification (oil immersion objective) using a Nikon Eclipse Ti2 microscope. Images were analyzed using Analyze Particles tool in ImageJ2 software [25].

### Lipidomics

Lipid analysis was performed at the Lipidomics Service, IQAC-CSIC (Barcelona, Spain). One brain hemisphere was homogenized in sterile PBS using a TissueLyserII equipment (Qiagen) and protein concentration was determined by Bradford assay [26]. Lipid extraction, identification, and quantification by liquid chromatography coupled with high-resolution mass spectrometry (LC–MS/ToF) were performed as described [14,21]. The analysis identified a total of 169 lipid species in the brain samples that were annotated according to LIPID Metabolites and Pathways Strategy (LIPID MAPS) indications as described in [26] and including the following: Sphingolipids (GM1, ganglioside 1 and GM3, ganglioside 3) and Glycerophospholipids (PG, phosphatidylglycerol). Lipidomics data were analyzed with web-based Metaboanalyst 6.0 [27] as described in [26] to create heatmaps showing normalized, log_2_-transformed, pareto-scaled data and displaying the top 25 lipid features selected by hierarchical clustering as previously mentioned in [21].

### Transcriptomics

Reanalysis of RNA-seq data from mice infected with WNV *vs* uninfected mice in brain and cerebellum tissues was performed with GEO datasets GSE242979**,** GSE233216 and GSE233217**.** For transcriptomic analysis of mice treated with A922500, RNA extraction and cDNA library preparation was performed as described [20]. Sequencing was performed on a NovaSeqX platform (Illumina, San Diego, USA) by Macrogen (Seoul, Korea). Quality control and bioinformatic raw data preprocessing were performed by Macrogen [20] to obtain differentially expressed genes (DEGs). The entire DEGs dataset ranked by log_2_fold change was analyzed with STRING [28] for Gene Set Enrichment Analysis (GSEA).

### Quantitative RT–PCR

Total RNA was extracted from brain samples using Ribopure (Invitrogen). Viral burden was determined by Real-time RT–PCR [29] and was expressed as cycle threshold (Ct) values. Real-time RT–PCR was performed for 40 amplification cycles. Ct values ≥40 were considered negative [30].

### Cytokines in the brain

Brain samples were homogenized using Tissuelyser II (5 min, 25 Hz) in the presence of 500 µl/100 mg of Cell Extraction Buffer (Invitrogen) supplemented with 0.5 M EDTA, and with Halt Protease and Phosphatase Inhibitor Single-Use Cocktail (Invitrogen). Samples were centrifuged at 16000 x g for 10 min at 4 °C, and protein concentration was measured using Bradford assay and standardized to 10 mg/ml. Mouse cytokines and chemokines were quantified using a multiplex fluorescent bead immunoassay (ProcartaPlex Mouse Th1/ Th2 chemokine panel 20 plex; ThermoFisher) and a MAGPIX system (Luminex Corporation). Concentrations were obtained using xPonent version 3.1 software based on standard curves provided by the manufacturer.

### Data analysis

In line and bar graphs, data are presented as mean ± SEM. In box and whisker plots, cages indicate median ± quartile, and whiskers expand to the maximum and minimum values. Dose response curves were adjusted by nonlinear regression using a log (inhibitor) *vs* normalized response (variable slope) equation using PRISM GraphPad software. Details of the statistical analyses are provided in the figure legends.

## Results

### Remodelling of mice brain lipidome upon WNV infection

**Figure 1.**
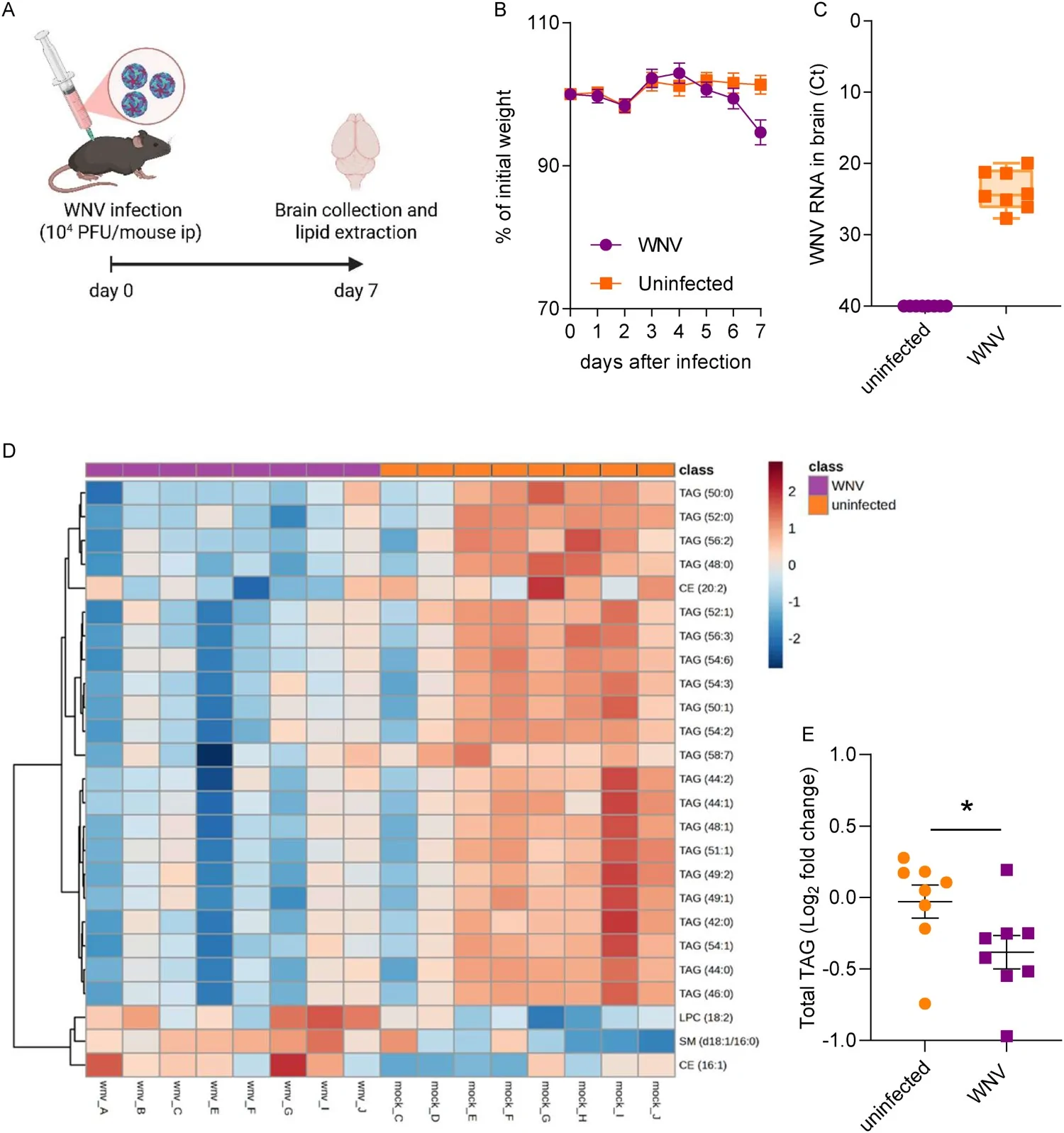
Remodelling of mice brain lipidome upon WNV infection. (a) Experimental design. C57BL/6 female mice were infected with WNV (10^4^ PFU/mouse ip) or mock-infected (uninfected), and all animals were euthanized at 7 dpi. Brains were harvested and processed for RNA extraction to determine viral burden and lipid extraction to explore changes in the lipidome. Figure was created with Biorender. (b) Change in mouse body weight after WNV infection (n =  8 animals per group). Data is presented as mean ± SEM. (c) Virus burden in the brain determined by quantitative RT-PCR in infected and uninfected mice euthanized at 7 dpi. (n =  8 animals per group). Boxes indicate median ± quartile and whiskers expand to maximum and minimum values. Ct values ≥40 were considered negative. (d) Heatmap showing the top 25 lipid species features selected by hierarchical clustering and based on T-test/ANOVA. The distance measure was set to “Euclidean” and the clustering algorithm was set to “Ward.” Lipid levels on the heatmap denote normalized, log_2_-transformed fold change and Pareto-scaled values, with the degree of change marked with red (up-regulation) and blue (down-regulation). Each column denotes a single animal (*n* = 8 uninfected and *n* = 8 WNV infected mice at 7 dpi) and each row indicates a different lipid species. (e) Comparison of total TAG levels calculated as the sum of all the species identified in each animal between uninfected and infected brains. (n =  8 animals per group). Data is presented as mean ± SEM. * *p* < 0.05 for two-tailed unpaired Student’s t-test.

Six-week-old C57BL/6 female mice were infected with the highly neurovirulent WNV NY99 isolate (Figure 1(a)). Infected animals started to lose weight at day 7 post-infection (dpi) (Figure 1(b)). This time point constitutes an acute infection stage in which the virus has invaded the CNS (Figure 1(c)). Consistently, both infected mice and uninfected controls were euthanized at this time point to alleviate suffering by preventing the development of severe signs of disease and to anticipate the disease-induced death. Lipids were extracted from infected and uninfected brains and analyzed by liquid chromatography coupled with high-resolution mass spectrometry (LC-ToF). A total of 169 lipid molecular species spanning 4 categories (sphingolipids, glycerophospholipids, glycerolipids, and sterol lipids) and 16 subclasses of lipids were identified and quantified (Fig. S1). The analysis of the top ranked 25 molecular species showed that the main lipid species altered in infected brains in comparison to uninfected controls were TAGs (21/25) (Figure 1(d)). This alteration in total TAG content was also confirmed at the lipid subclass level (Figure 1(e)). Overall, these results indicated that infection by WNV remodeled the brain lipidome, particularly affecting TAG contents.

### WNV infection modulates the expression of TAG metabolism-related factors in the CNS

**Figure 2.**
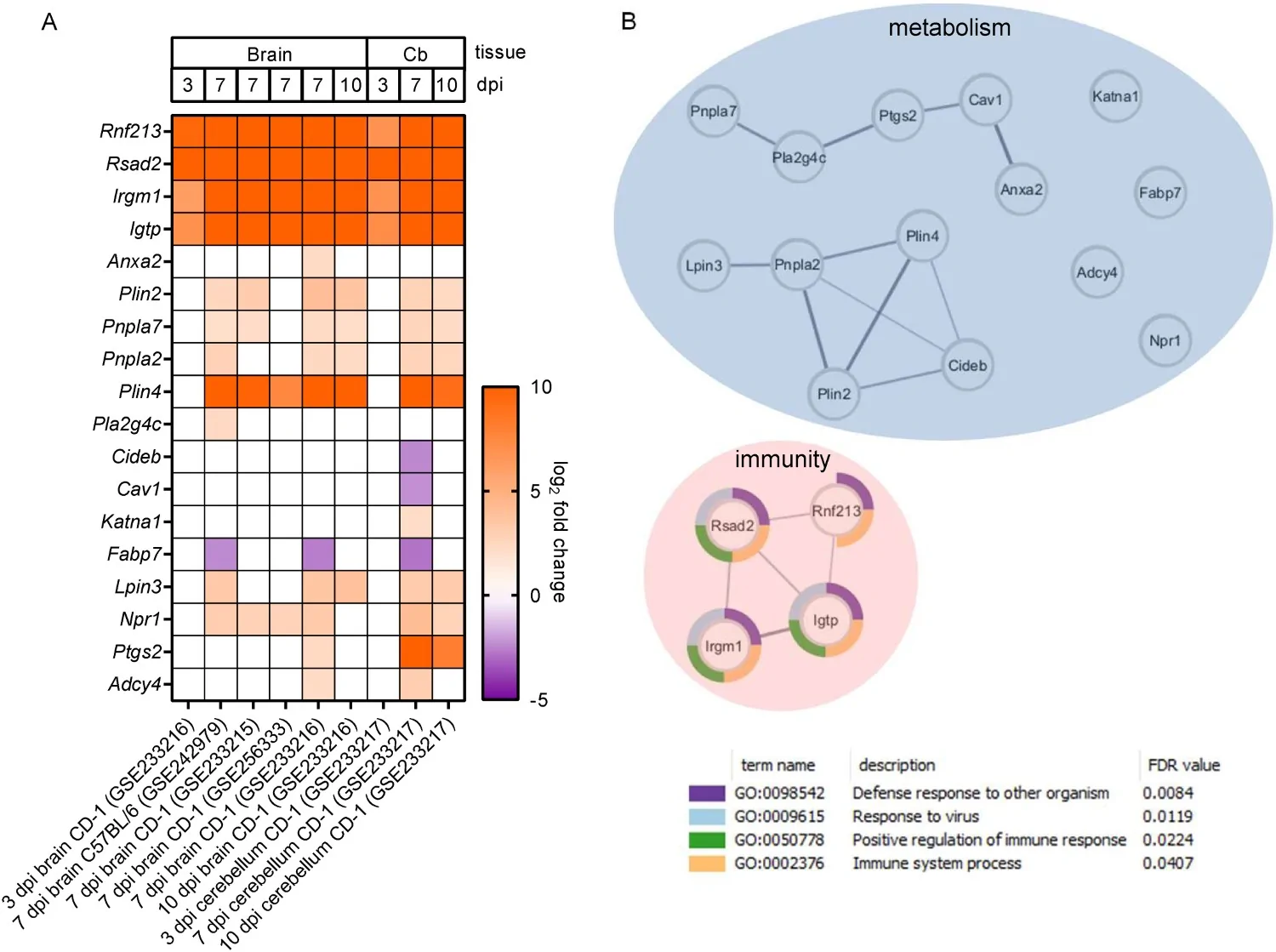
WNV infection modulates the expression of TAG metabolism-related factors in the CNS. (a) Expression patterns of DEGS related to TAG metabolism (Reactome R-MMU-8979227.1), lipid droplet (GO:0005811) and lipolysis (KEGG pathway mmu04923) in the CNS of WNV-infected mice. DEGs in the brain and cerebellum (Cb) between infected and uninfected mice treated in parallel were identified by reanalysis of publicly available transcriptomes in the GEO database (GSE233216, GSE242979, GSE233215, GSE256333, GSE233217). Heatmap colours denote gene expression values as Log2 fold change infected over uninfected. DEGS were included on the basis of |log2 foldchange|>2 and FDR-corrected *p*-value <0.05. (b) PPI networks within DEGs identified in panel (a). GO terms related to immune response identified by STRING enrichment in the subnetwork are indicated with its correspondent *p*-value FDR corrected.

TAGs are important storage lipids and the main components of cellular lipid droplets (LDs) [31]. Thus, the expression profile of genes related to TAG metabolism (R-MMU-8979227.1), LD (GO:0005811), and lipolysis (mmu04923) was examined across publicly available transcriptome datasets. This search identified a total of 18 differentially expressed genes (DEGs) from multiple datasets, covering different times post-infection (3, 7 and 10 dpi), mice strains (inbre d C57BL/6 and outbred ICR CD-1), and CNS structures (brain and cerebellum) (Figure 2(a)). Remarkably, the overexpression of perilipins (Plin2 and Plin4), phospholipases (Pnpla2, Pnpla7 and PLa2g4c), and lipin (Lipin3), together with downregulation of Cideb and Fabp7, was observed. Protein–protein interaction network analysis revealed a cluster enriched with immune-related processes, including defence response and response to infection (Rsad2, Rnf213, Irgm1, and Igtp) (Figure 2(b)). Overall, these results suggest that WNV infection rewired LD/TAG metabolism in the CNS with potential implications for viral multiplication and immune response.

### Relevance of cytoplasmic LDs for WNV infection

**Figure 3.**
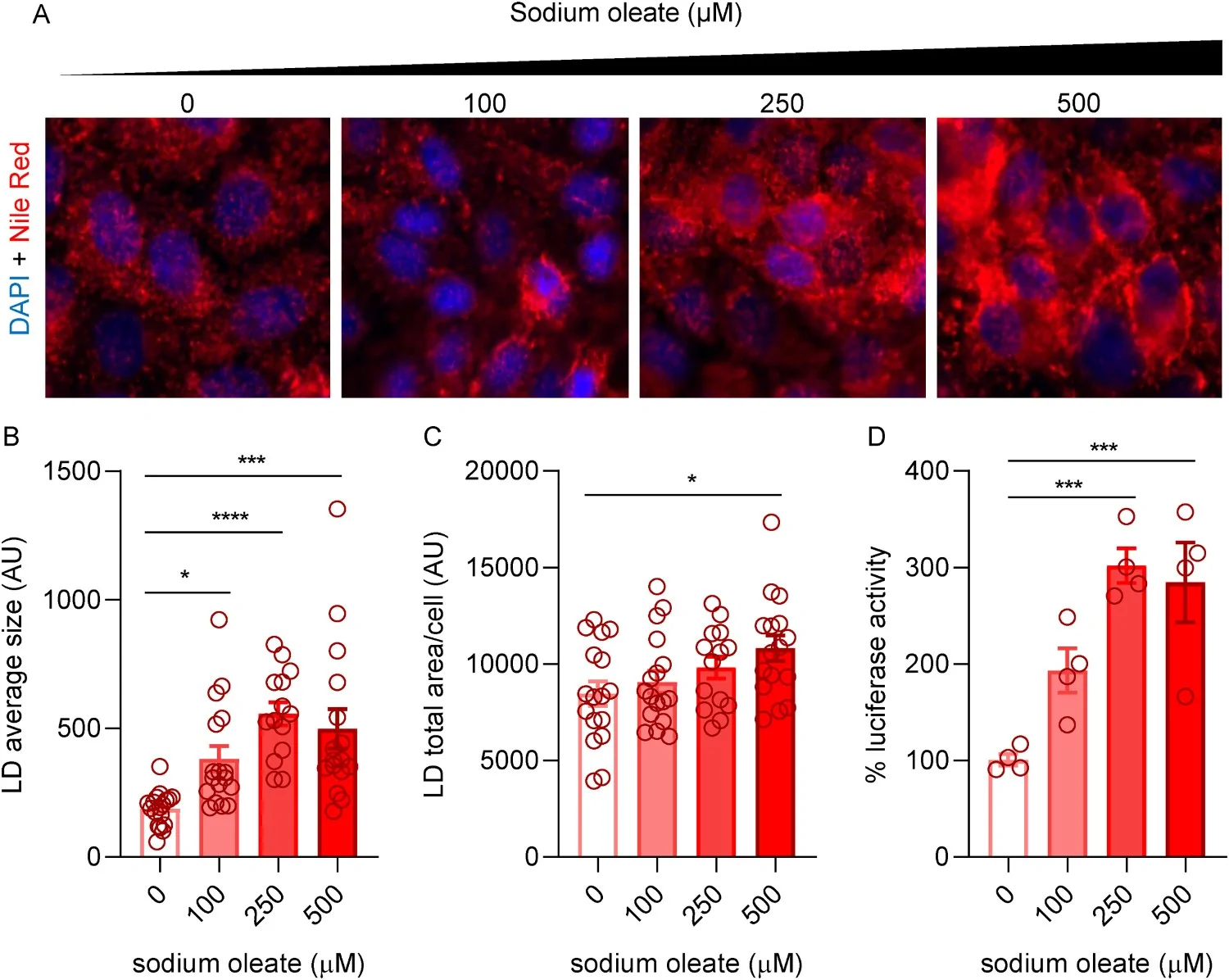
Relevance of cytoplasmic LDs for WNV infection. (a) Vero cells were pre-treated with different amounts of sodium oleate for 24 h and lipid droplets were stained with Nile Red (red). Cell nuclei were stained with DAPI (blue). (b and c) Quantification of LD average size (b) and LD total area/cell (c) in cells treated as in (a) (n = 14–18 cells per condition). (d) Effect of sodium oleate on the infection of WNV RVPs expressing luciferase. Cells were treated with increasing amounts of sodium oleate 24 h before infection and infected with RVPs. Lluciferase activity was quantified at 48 h post-infection. Data in (b, c, and d) are expressed as mean ± SEM. Asteriks denote * *P* < 0.05; *** *P* < 0.0005; and **** *P* < 0.0001 for two-tailed t-test with Dunnett’s correction for multiple comparisons.

As LDs are the main places of TAG accumulation, we analyzed their potential role in WNV infection. Supplementation of Vero cells with an exogenous source of fatty acids (sodium oleate) promoted a dose-dependent increase in cellular LD content as observed by Nile Red staining (Figure 3(c)), exhibiting an increase in cellular LD size (Figure 3(b)) and LD total area/cell (Figure 3(c)). To test the effect of this increase in LD content on WNV infection, we used single-cycle recombinant subviral particles (RVPs) as a surrogate model of infection. These infectious particles were produced by *trans*-complementation of a luciferase-encoding WNV replicon with a plasmid expressing WNV structural proteins. Treatment with sodium oleate promoted a dose-dependent increase in luciferase activity, with statistically significant differences between untreated cells and those treated with 250 and 500 µM (Figure 3(d)). Overall, these results are consistent with cytoplasmic LDs acting as important contributors to WNV multiplication.

### Antiviral activity of TAG inhibitors against WNV in cultured cells

**Figure 4.**
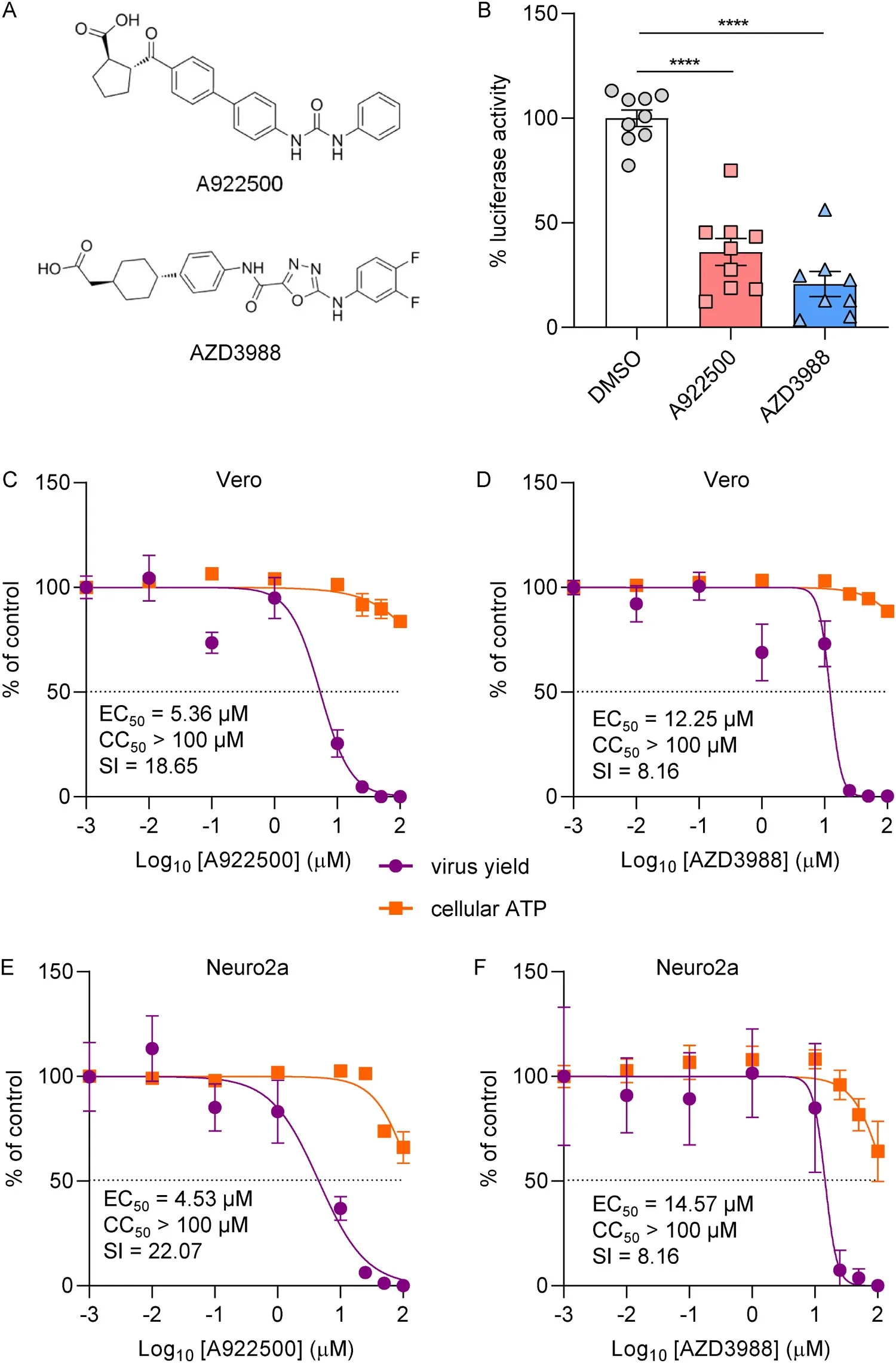
Antiviral activity of TAG synthesis inhibitors against WNV in cultured cells. (a) Chemical structure of inhibitors used in this study. (b) Effect of DGATi on the infection of WNV RVPs expressing luciferase. Cells were infected with RVPs and treated with 50 µM of DGATi, and luciferase activity was quantified at 48 h post-infection. **** denote *P* < 0.0001 for two-tailed t-test with Dunnett’s correction for multiple comparisons. (c–f) Antiviral activity against WNV and cytotoxicity of DGATi in Vero cells. The virus yield in the supernatant of infected Vero (c, d) and Neuro2a (e, f) cells (MOI of 1 PFU/cell) was determined at 24 h p.i. DGATi were added 1 h before infection and maintained throughout the rest of the assay. The cytotoxicity of the compounds was measured by the determination of the cellular ATP concentration in uninfected samples. (e, f) Antiviral activity against WNV and cytotoxicity of DGATi in Neuro2a cells were determined as in panels (c) and (d). The dashed lines denote a 50% reduction.

Given the strong association between LDs and TAGs, we analyzed the potential effect of TAG synthesis inhibitors on WNV infection. Diacylglycerol acyltransferases (DGAT-1 and 2) catalyze the last step in TAG biosynthesis by esterifying diacylglycerol with fatty acyl-CoA [32], making them not only key metabolic regulators but also promising therapeutic targets. Among the DGAT inhibitors (DGATi) described, we selected two commercially available DGAT-1 inhibitors: A922500 [33] and AZD3988 [34] (Figure 4(a)). Treatment of Vero cells with 50 µM of both compounds reduced luciferase expression following infection with RVPs, which was compatible with a reduction in viral multiplication (Figure 4(b)). Additional experiments involving live WNV showed a dose-dependent reduction of the multiplication of WNV, with half-maximal effective concentrations (EC_50_) of 5.36 and 12.25 µM for A922500 and AZD3988, respectively (Figure 4(c) and (d)). Half-maximal cytotoxic concentrations (CC_50_), estimated by cellular ATP measurement, were >100 µM for both compounds, indicating that antiviral activity was not the result of cytotoxic effects. This resulted in selectivity indexes (SI), calculated as the ratio between CC_50_ and EC_50_, of 18.65 and 8.16 for A922500 and AZD3988, respectively. Similar results were obtained in Neuro2a cells, a cell line from neuronal origin, obtaining EC_50_ values of 4.53 and 14.57 µM for A922500 and AZD3988, respectively (Figure 4(e) and (f)). Regarding cytotoxicity of the compounds, CC_50_ > 100 µM were also calculated for both compounds in Neuro2a cells, giving an SI of 22.07 and 6.86 for A922500 and AZD3988, respectively. This antiviral activity observed in cell cultures, together with the low impact on cell viability, supports the antiviral potential of DGAT inhibitors against WNV.

### Mechanism of DGATi-mediated suppression of WNV infection

**Figure 5.**
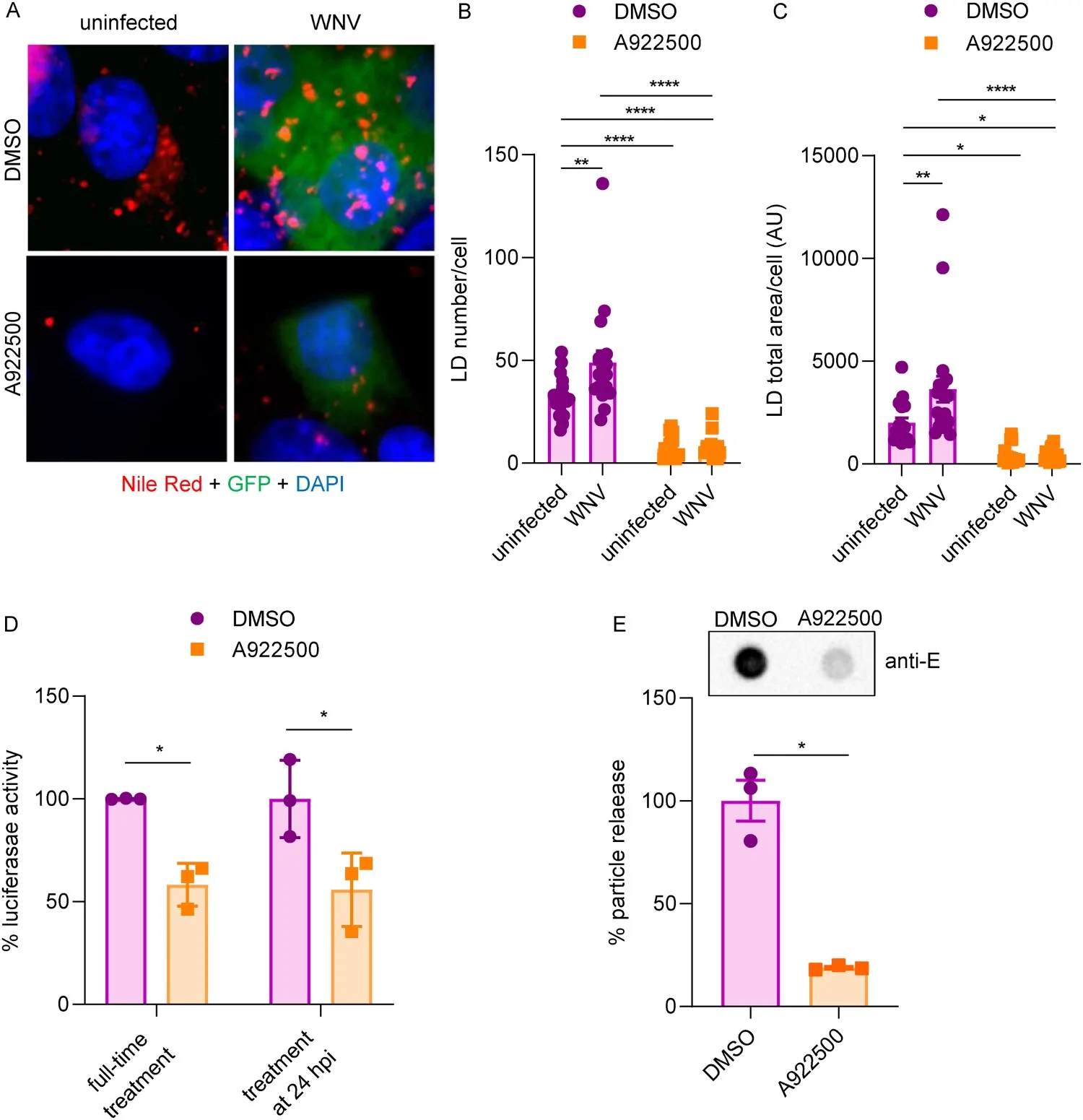
Mechanism of DGATi-mediated suppression of WNV Infection. (a) Representative fluorescence micrographs of Vero cells were treated with 50 µM A922500 and infected with GFP-expressing RVPs (green) at 48 hpi. Lipid droplets were stained with Nile Red (red), and cell nuclei were counterstained with DAPI (blue). Uninfected cells and vehicle-treated control cells are included. (b, c) Quantification of the number of LDs (b) and total LD area (c) in Vero cells upon treatment with 50 µM A922500 as shown in panel A. Each symbol represents a single cell analyzed (n = 16–19). Data are presented as mean ± SEM. Asteriks denote * *P* < 0.05; ** *P* < 0.005; and **** *P* < 0.0001 for ANOVA using Tukey's correction to adjust for multiple comparisons. (d) Effect of A922500 on the infection of WNV RVPs expressing luciferase. Cells were infected with RVPs and treated with 50 µM of A922500, and luciferase activity was quantified at 48 h post-infection. Full-time treatment indicates that A9225000 was added 1 h before infection and was maintained throughout the experiment, whereas treatment starting 24 hpi indicated that the compound was added after 24 h of RVP infection and maintained throughout the rest of the assay (n =  3). Data is presented as mean ± SEM. Asterisks denote * *P* < 0.05; for ANOVA using Tukey's correction to adjust for multiple comparisons. (e) Hela cells stably expressing WNV prM and E were treated with 50 µM A922500 or DMSO control. Culture supernatants were collected, and the amount of released VLPs was determined by immunoblot analysis as described in the Materials and Methods section. The graph shows the quantification of extracellular VLP production relative to the vehicle-treated control (n = 3). Data are presented as mean ± SEM. * denote for *P* < 0.05 for two-tailed t-test with Welsch’s correction.

We further investigated the antiviral activity of A922500 as a model DGATi. First, the impact of A922500 on intracellular LD content was assessed by Nile Red staining on uninfected cells and on cells infected with GFP-expressing RVPs (Figure 5(a)). A similar reduction in the number of LDs (Figure 5(b)) and LD total area /cell (Figure 5(c)) was observed due to the treatment with 50 µM A922500 in both uninfected and RVP-infected cells. These results confirmed that the compound blocked LD formation in both infected and uninfected cells. To dissect the stage of WNV life cycle affected by DGATi, time-of-addition experiments were carried out using single-cycle RVPs expressing luciferase reporter (Figure 5(d)). Results showed that 50 µM A922500 inhibited luciferase expression even when administered 24 h post-infection. Notably, the magnitude of inhibition was comparable to that observed when the compound was added 1 h before infection and maintained throughout the experiment. These findings are consistent with A922500 targeting processes associated with viral replication rather than early steps of infection. Given the close relationship between WNV replication, virion assembly, and host lipid metabolism, we next examined whether DGAT inhibition may also affect viral morphogenesis. For this purpose, we employed a cell line that constitutively expresses WNV structural proteins and continuously releases virus-like particles (VLPs) independently of viral genome replication [24]. Immunoblot analysis of culture supernatants demonstrated a significant reduction in VLP production following treatment with 50 µM A922500 (Figure 5(e)). Collectively, these data indicate that DGAT inhibition disrupts not only WNV replication but also the assembly and/or release of viral particles, highlighting the importance of DGAT for varied steps of the WNV life cycle.

### *In vivo* efficacy of a DGATi against WNV

**Figure 6.**
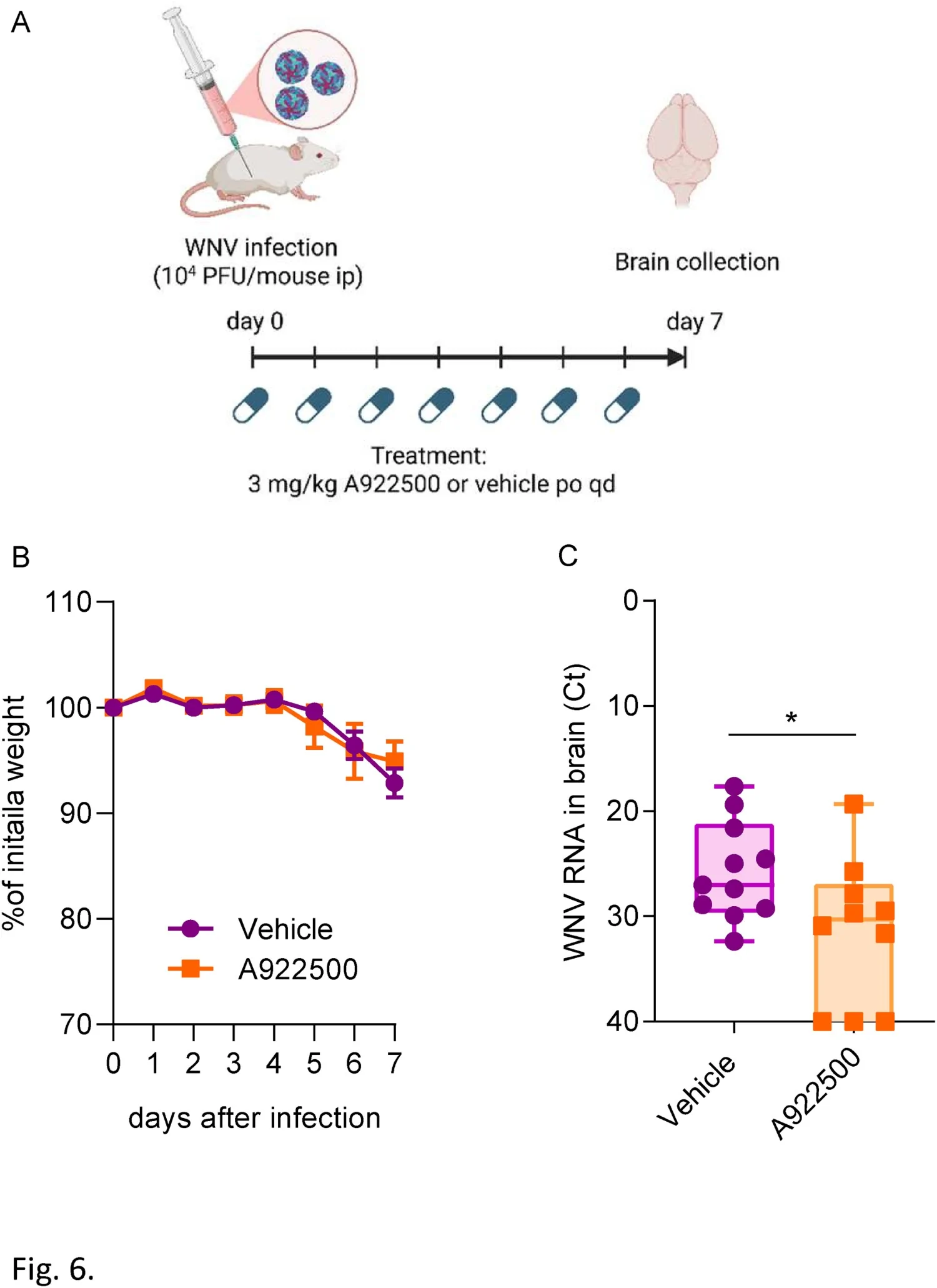
In vivo efficacy of a DGATi against WNV. (a) Experimental design. CD-1 female mice were infected with WNV (10^4^ PFU/mouse ip) or mock-infected (uninfected), and all animals were euthanized at 7 dpi. A group of mice was treated with A922500 once a day po, and control infected mice were treated in parallel with compound vehicle alone. All mice were euthanized at 7 dpi, and brains were harvested for analyses. Figure was created with Biorender. (b) Change in mouse body weight (n =  11 animals per group). Data is presented as mean ± SEM. (c) Virus burden in the brain determined by quantitative RT-PCR in infected and uninfected mice euthanized at 7 dpi. Each symbol denotes a single animal (n =  11 animals per group). Boxes indicate median ± quartile and whiskers extend to maximum and minimum values.

From the two DGATi tested, A922500 was selected for in vivo efficacy studies in the mouse model of WNV infection. Importantly, A922500 provided the best results in the cell culture assays and, more relevantly, its beneficial effects have been demonstrated in preclinical studies involving CNS disorders [35,36]. Six-week-old CD-1 female mice were infected with WNV and treated orally with the compound or its vehicle as a control (Figure 6(a)). A similar reduction in body weight was observed for both groups (vehicle and A922500) of mice (Figure 6(b)). Thus, both groups of infected mice were euthanized at 7 dpi to maximize their well-being and minimize the severity of the procedure (Figure 6(b)). Brains were harvested and viral burden in brain was quantified by real-time PCR (Figure 6(c)). A significant reduction in viral burden was observed in the group of mice treated with A922500 when compared to vehicle group (Figure 6(c)), confirming that DGAT inhibition exerts an antiviral effect *in vivo*.

### Impact of A922500 in the brain transcriptome of infected mice

**Figure 7.**
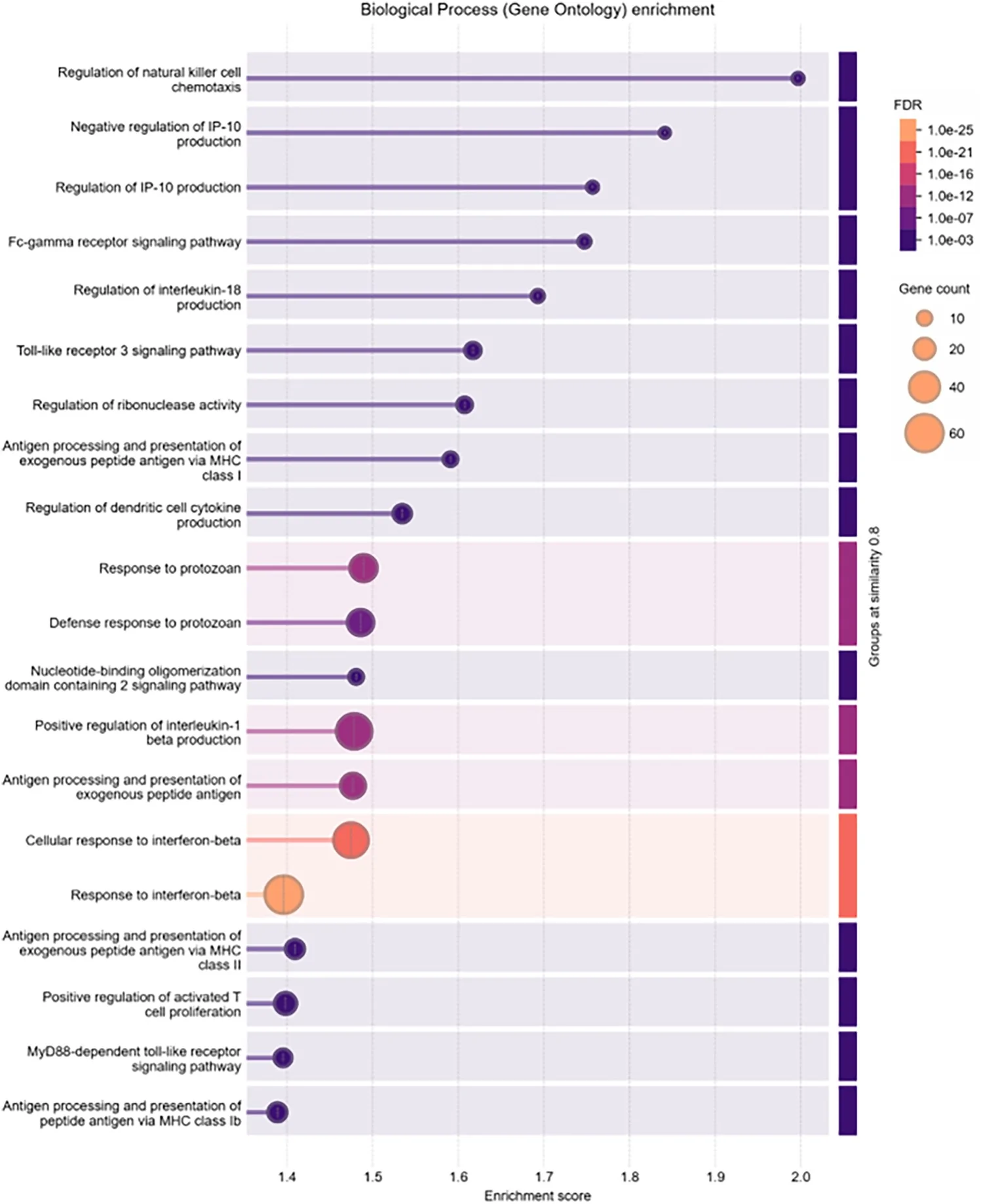
Impact of A922500 in the brain transcriptome of infected mice. Gene set enrichment analysis of biological processes in the brain of mice treated with A922500 compared to vehicle-treated mice was performed with STRING.

To provide further insights into the effect of A922500 on the infected brains, a transcriptomics approach was followed. To this end, bulk RNA-sequencing (RNA-seq) was conducted in infected mice treated with A922500 or vehicle alone. The transcriptional rearrangements between the brains of infected mice treated with A922500 in comparison to infected vehicle-treated mice alone were explored using GSEA [37]. This analysis identified the biological pathways that were most significantly altered by treatment (Figure 7). Most of the top significantly enriched pathways identified were related to immune responses, encompassing innate, cellular, and humoral immunity. Specifically, the analysis identified pathways related to antiviral sensing through toll-like receptor 3 (TLR3), IFN-β responses, cytokine production (including IP-10/CXCL10, IL-18, and IL-1β), regulation of natural killer cell activity, antigen processing and presentation, and T-cell proliferation. Together, these transcriptomic changes are consistent with a broad immunomodulatory effect of A922500 in the brains of WNV-infected mice.

### Effect of A922500 on cytokine induction in brain

**Figure 8.**
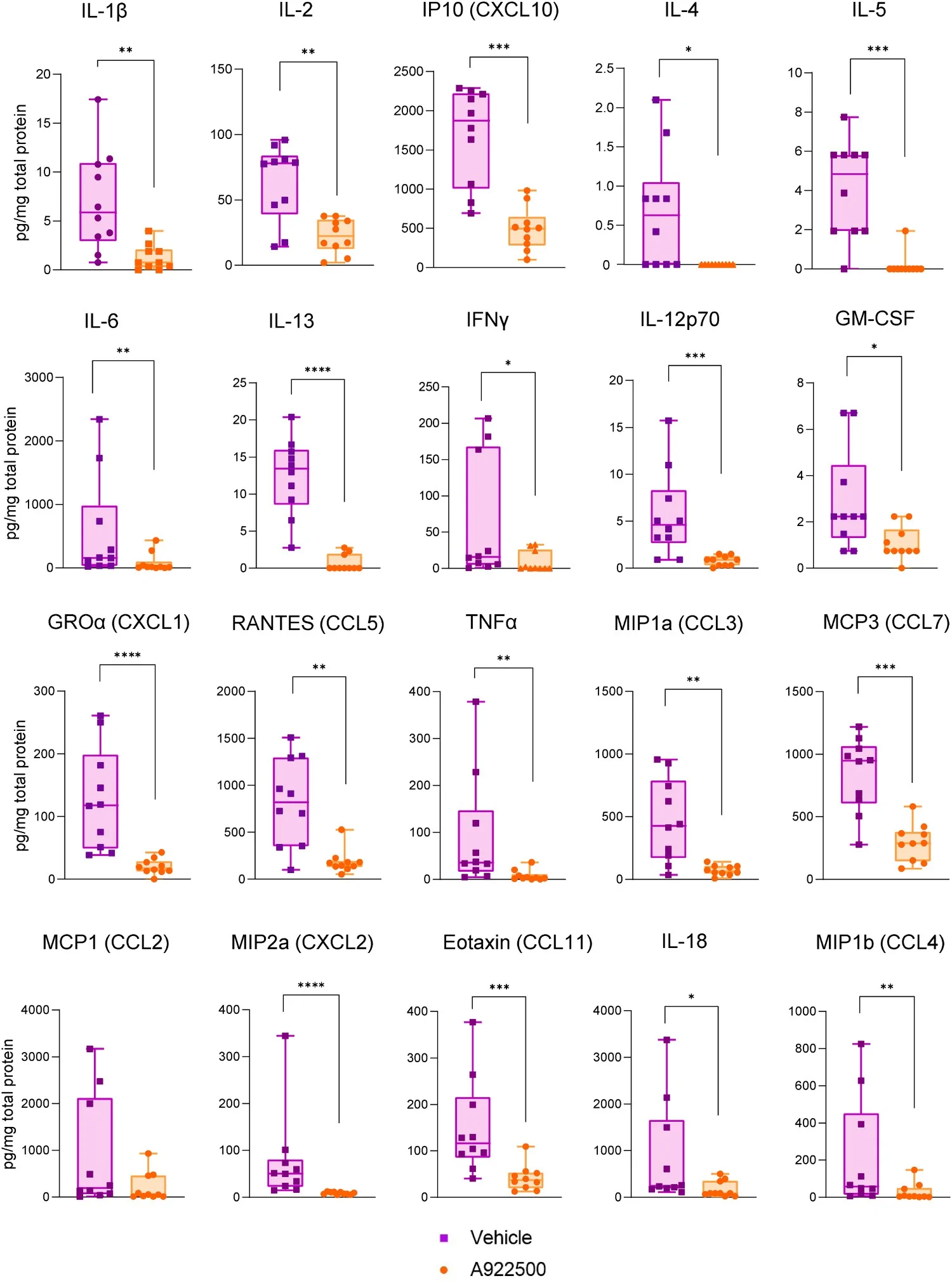
Cytokine profiles in treated mice. Quantification of cytokine content in WNV-infected mice treated with A922500 or its vehicle measured by Luminex. Values are shown as pg of cytokine normalized by mg of total protein, and each symbol denotes an animal. Group means are indicated by lines, and error bars denote SEM. *p < 0.05; **p < 0.01; ***p < 0.001; ****p < 0.0001 for Mann-Whitney test.

The effect of the compound on the production of cytokine/inflammation mediators in the brains of WNV-infected mice was further analyzed using a multiplex fluorescent bead immunoassay (Figure 8). Our results showed a significant reduction in the expression of cytokines associated with pro-inflammatory immune responses (IL-6, IL-1β, IL-12 p70, IL-18), Th1 cytokines (TNF-α, IL-2, IFN-γ), Th2 (IL-4, IL-5, IL-13), and chemokines (Eotaxin (CCL11), GROα (CXCL1), IP10 (CXCL10), MCP3 (CCL7), MIP-1α (CCL3), MIP-1β (CCL4), MIP2α (CXCL2), RANTES (CCL5)), and a tendency toward reduced MCP1 (CCL2) expression. Overall, these results indicate that A922500 attenuates WNV-induced neuroinflammatory responses in the murine brain.

## Discussion

The study of the cellular biology of WNV infection in cultured cell models has unveiled the importance of lipid metabolism for the virus life cycle [10,14]. Different studies have confirmed the *in vivo* relevance of specific lipids in WNV infection. These findings include remodelling of blood lipid content in mice and WNND patients [21,38], changes in the lipid content in the liver of infected mice [26], and highlight the importance of certain sphingolipids for multiplication and neuroinflammation [18,39]. Nevertheless, important gaps remain in translational research needed to translate these findings into effective therapies. To identify the lipid pathways with potential therapeutic targets, we investigated the changes induced by infection in the lipidome of the CNS, which is the main target tissue for WNV replication. Our findings revealed that the predominant changes in brain lipid composition in WNV infected mice were attributable to alterations in TAGs, a subclass of neutral lipids that perform important physiological functions, serving as the major storage molecules of metabolic energy and fatty acids, regulating cell signalling, immune function, and inflammation [40].

The here described impact of WNV infection on TAGs is consistent with the importance of LDs in orthoflavivirus infection [41]. Thus, the reduction of TAGs found in infected brains can point to efficient breakdown or export of TAGs from the LDs to fuel viral replication and tissue response to infection. The analysis of the brain and cerebellum transcriptome from WNV-infected mice confirmed that the infection by WNV altered the expression patterns of a variety of factors related to TAG metabolism and LD function in the CNS. These DEGs included LD-coating perilipins (Plin2 and Plin4), which regulate TAG storage by controlling lipase access to the droplet surface [42]. Remarkably, Pnpla2 (also known as adipose triglyceride lipase, Atgl), the major TAG lipase on LD catalyzing the rate-limiting step of TAG hydrolysis and supplying fatty acids for cellular energy demands [43], was also upregulated across different samples. Pnpla7, which also contributes to lipid remodelling and indirectly influences TAG metabolism and LD dynamics, was similarly upregulated [44]. Downregulation of Cideb and Fabp7, also realted to TAG and LD metabolism [45,46] were consistent with the alteration of TAG content in infected brains.

TAG metabolism is implicated in a broad range of metabolic disorders, including obesity, diabetes, non-alcoholic fatty liver disease, steatohepatitis, and atherosclerosis. Moreover, dysregulation of TAG metabolism contributes to an elevated risk of cardiovascular disease. Together, these findings highlight TAG metabolism as an important therapeutic target for drug development [47-49]. Among DGAT inhibitors commercially available, we selected DGAT-1 inhibitors over DGAT-2 inhibitors due to their proven efficacy for the treatment of CNS-related diseases in animal models, including viral neuroinflammation [35,47] and autoimmune encephalitis [36]. Thus, we tested the antiviral activity of A922500 and AZD3988 in Vero and Neuro2a cells. Both compounds reduced WNV multiplication at concentrations that did not compromise cell viability. Given the superior potency of A922500 against WNV in cell cultures, together with its good safety record in animal experiments for the treatment of a wide variety of disorders [35,36,50-52], this compound was evaluated in *in vivo* efficacy assays. Our results indicated a reduction in the viral burden in the brain of treated mice, providing a proof-of-concept of the antiviral efficacy of this approach *in vivo*.

Furthermore, our results indicate that A922500 modulates the immune environment of WNV-infected brains by broadly attenuating inflammatory mediators, which may result from direct anti-inflammatory effects of DGATi and/or from the reduction in viral burden. We noticed a broad decrease in pro-inflammatory cytokines and Th1- and Th2-associated cytokines, together with a reduction in chemokines involved in leukocyte recruitment to the CNS, including those that attract myeloid cells (CCL11, CXCL1, CCL7, CCL3, CCL4, CXCL2, CCL5, and a tendency in CCL2) and lymphocytes (CXCL10). Moreover, in the context of WNV infection, where inflammation is necessary for viral clearance but simultaneously contributes to bystander neuronal injury, attenuation of pro-inflammatory pathways has become a key mechanism for limiting neuropathology. For example, dysregulated or sustained IL-1β signalling has been associated with chronic neuroinflammatory damage, resulting in persistent cognitive impairment in a WNND model [53], providing a mechanistic link between IL-1β-mediated inflammation and long-term neurological sequelae. Elevated levels of IL-6 have also been associated with neuroinvasive disease and persistent inflammatory features [54]. Similarly, other pro-inflammatory cytokines (including IL-12p70, IL-2, IFN-γ, and the chemokine IP-10) have also been related with prolonged post-WNV symptomatology in humans [55]. Regarding the role of chemokines, CCL7 and CCL2 promote the recruitment of inflammatory monocytes to the CNS [56,57], but notably, CCL2 neutralization improves survival in WNV-infected mice [57]. CXCL10 also appears to have a dual biological role, as excessive levels of this chemokine have been linked to immunopathology due to its ability to induce neuronal apoptosis [58]. Additionally, increased infiltration of CD8+ T cells in the CNS has also been associated with enhanced tissue injury during WNV encephalitis. Taken together, the broad reduction in these mediators supports an attenuation of WNV-induced neuroinflammation.

Transcriptomic data also revealed modulation of broader immune pathways (including innate sensing, interferon-regulated programmes, antigen presentation, and lymphocyte-associated processes) suggesting that DGAT inhibition may reshape the neuroimmune environment beyond the specific cytokines quantified, further aligning with the overall attenuation of neuroinflammation observed in treated brains. The observed reduction of viral burden in the brain as well as neuroinflammation is consistent with the dual role of TAGs fuelling viral replication and regulating immune response observed in other viral models [35]. These results support previous reports describing the beneficial therapeutic effects of A922500 in modulating ZIKV-induced neuroinflammation [35,47] and autoimmune encephalitis [36]. Although treatment with A922500 significantly reduced brain viral burden and inflammatory cytokine levels, the absence of an effect on body weight in WNV-infected mice during the monitoring period suggests that optimization of the treatment regimen (including dose and timing) may be required before a meaningful survival benefit can be expected. Future studies incorporating additional clinical endpoints, including survival, will be important to further evaluate therapeutic efficacy and enhance the translational potential of these findings. Moreover, sustained DGAT-1 inhibition has been associated with gastrointestinal side effects [59]. Nevertheless, it remains to be determined whether currently available or future developed DGAT-1 inhibitors might hold therapeutic value in short-term treatments, as would be expected for the acute phase of WNV infection.

Overall, our results unveil the importance of glycerolipid metabolism for WNV and uncover the potential of therapeutic interventions on this pathway for the control of viral replication and neuroinflammation. Thus, TAGs could represent a promising dual-action therapeutic target for both antiviral and anti-inflammatory interventions against WNND.

## Supplementary Material

graphical_abstract_A922500_525px_width.PNG

Supplementary figure legend.docx

Supplementary figure 1.tiff

## Data Availability

The dataset supporting the conclusions of this article is available in the Gene Expression Omnibus repository under accession GSE310714.
