# Supplementary material for: Triacylglycerol metabolism is a novel target to combat West Nile virus infection
Source: Emerg Microbes Infect. 2026 Sep 1;15(1):2728216. doi: 10.1080/22221751.2026.2728216 (PMC13591993; doi:10.1080/22221751.2026.2728216)
Supplement: Supplementary file 1 [file TEMI_A_2728216_SM0160.docx]

**Supplementary material**

**Figure S1. Heatmap of the lipidome of the brain from mice infected with WNV**. Lipid levels in the scales denote normalized, log_2_-transformed fold change and Pareto-scaled values. Columns denote analyzed animals (n = 8 mice/group) and rows lipid species ordered by subclass and total carbon. Samples were grouped by hierarchical clustering.
